# PACT: A practice-driven predictive algorithm for customized transradial prosthetic socket design

**DOI:** 10.1371/journal.pone.0340831

**Published:** 2026-01-08

**Authors:** Vishal Pendse, Calvin C. Ngan, Elaine Ouellette, Neil Ready, Jan Andrysek

**Affiliations:** 1 Institute of Biomedical Engineering, University of Toronto, Toronto, Ontario, Canada; 2 Holland Bloorview Kids Rehabilitation Hospital, Toronto, Ontario, Canada; University of Illinois Urbana-Champaign, UNITED STATES OF AMERICA

## Abstract

Well-fitting sockets are crucial for successful upper-limb prosthesis use, yet current digital socket design workflows are not standardized and demand considerable clinician effort. In this study, we introduce the Predictive Algorithm for Customized Transradial Socket Design (PACT), which generates socket models from a 3D limb scan. It works by retrieving the most similar limb–socket pair from a reference library of prosthetist-designed prosthetic sockets and applying isotropic and anisotropic scaling adjustments to match the input limb. To validate the algorithm, the PACT-predicted sockets for 19 participants were compared to their prosthetist-designed ones (the clinical “gold standard”) using the surface Euclidean (L2) distances, volume differences, and a 100-slice cross-sectional-area analysis. Localized discrepancies were mapped via signed-distance colorization and clustered with DBSCAN. PACT’s outputs differed from prosthetist designs by 2.11 ± 0.51 mm on the surface and 2.74 ± 2.56% in volume on average; slice-wise area differences were within ±10% for most of the socket length, with larger errors near the proximal trimline and distal tip. Recurrent localized discrepancies were found to be concentrated at the anterior-distal trimline (15/19 cases) and anterior–posterior compression (11/19 cases), indicating clear targets for rule-based or measurement-informed refinements. Subgroup patterns suggested an age-related bias (undersizing in pediatric, oversizing in adults). Overall, PACT quickly delivers (13.2 ± 0.7 s) a first-draft transradial socket within commonly cited clinical fit tolerances. Focus on specific regions, along with metadata such as tissue stiffness, age, and clinician-led measurements, can improve personalization and generalizability in future iterations of the PACT.

## Introduction

The prosthetic socket serves as a critical interface between a user’s residual limb and the prosthesis. Its design plays a key role in determining comfort, fit, and function, which, in turn, influence prosthesis effectiveness and patient outcomes [1]. Traditionally, socket fabrication has been a largely manual process, where prosthetists first capture the residual limb through plaster casting, followed by rounds of “rectification” to create a custom-fit socket [2]. These techniques are time-consuming, material-intensive, and highly dependent on individual expertise [3,4]. Recent advancements in digital technology promise to streamline this workflow: handheld 3D scanners can accurately and quickly capture limb geometry. CAD software supports the virtual rectification of these 3D models, and additive manufacturing can assure consistent fabrication [5–8]. However, translating hands-on knowledge, which informs socket comfort and biomechanics, into the digital realm is challenging and has raised the need for quantitative design approaches [9–12].

Several studies have shown promise in enhancing and standardizing prosthetic socket design. Li et al. leveraged the rectification patterns of three transtibial-socket prosthetists to derive quantitative “compensation” factors using an eigenvector algorithm, leading to reduced interface pressures and improved comfort in a ten-user study [13]. Steer et al. introduced two in-silico frameworks: one based on multi-objective genetic algorithms to generate Pareto-optimal transtibial sockets that redistribute loads from prominences [14], and another using finite element analysis-driven surrogate models to accurately predict stress and strain fields in real-time [15]. However, both were only tested with virtual limbs and did not produce sockets. In a different approach, Torres-Moreno et al. created a digital reference shape library of above-knee plaster casts, enabling clinicians to select a socket design based on simple anthropometric measurements, thus preserving expert-crafted geometry and significantly reducing early-stage workload [16]. Despite feasibility, adoption was limited by manual data entry and frequent model tweaks: issues potentially solvable through automation [16,17]. Additionally, none of the aforementioned methods have yet been applied to upper-limb prosthetics.

Transradial amputations account for 42% of all major upper-limb amputations (those proximal to the wrist) [18]. The design of transradial prosthetic sockets presents unique challenges due to their self-suspending nature, the absence of a pressure-relieving liner, and direct skin contact [19]. These factors necessitate nuanced, evidence-based solutions [20], such as a snug fit above the epicondyles for secure suspension, sufficient distal space to accommodate dynamic changes in limb volume, and growth (particularly in pediatric cases) [9,19,21]. High rates of upper-limb prostheses abandonment have been reported due to issues such as poor function and comfort, which are directly related to prosthetic limb and socket design [22,23]. This supports the need for advancement of transradial socket design approaches, and exploration of data-driven methods to quantify best clinical practices.

The goal of this work was to develop and evaluate the Predictive Algorithm for Customized Transradial socket design (PACT), a prosthetist-practice-driven algorithm for automating the selection and customization of transradial prosthetic sockets. PACT uses a library of existing prosthetist-created designs to automatically predict a customized socket from a 3D limb scan, which can ultimately be fabricated via additive manufacturing.

This study was structured around two sequential aims: The first aim is focused on algorithmic development. Specifically, objective 1a establishes a reference library comprising 3D scans of transradial residual limbs paired with their corresponding prosthetist-fabricated sockets. Objective 1b is to develop the PACT algorithm, which identifies the limb–socket pair in the library that most closely matches a new client’s scan and then customizes that socket to the client’s specific anatomy.

The second aim is to evaluate the algorithm. Specifically, this includes a global evaluation (Obj 2a) where the PACT-predicted sockets are compared to their prosthetist-fabricated counterparts using surface distances, volumes, and slice-wise cross-sectional areas. It also includes a local evaluation (Obj 2b) where local shape deviations are mapped and clustered to identify areas for future algorithmic refinement. Finally, a subgroup analysis examines whether participant-specific factors such as limb geometry and demographics influenced prediction results (Obj 2c).

## Methods

### Algorithm development—Obj 1

This section outlines the construction of the reference library and the development of the PACT workflow.

#### Data collection.

Nineteen (n = 19) participants with transradial upper-limb absence were recruited for this study, from January 21, 2020 to January 30, 2025. The inclusion criteria were as follows: a) participants must have had transradial limb absence for a minimum of two years, and b) a new prosthetic socket was required during the data collection period. Informed written consent was obtained from all participants in the study; for those under the age of 18 years, written guardian consent and participant assent were obtained. The study protocol was approved by the Research Ethics Board at Holland Bloorview Kids Rehabilitation Hospital (File #19–864; approved on 21 January 2020), in compliance with ethical standards for research with human participants. Table 1 shows the demographics of all participants, where qualitative limb descriptors were assessed by the treating clinician.

**Table 1:** Participant demographics.

				Qualitative limb descriptors	
Participant	Sex	Age (years)	Limb length (mm)	Length*	Shape**
P01	F	15	66.6	Short	Conical
P02	M	10	78.6	Very short	Cylindrical
P03	F	13	35.4	Short	Cylindrical
P04	F	22	45.8	Very short	Cylindrical
P05	M	17	46.4	Very short	Cylindrical
P06	F	16	75.6	Very short	Conical
P07	M	61	59.2	Very short	Conical
P08	M	47	78.4	Short	Conical
P09	M	55	87.9	Short	Conical
P10	F	29	88.1	Short	Conical
P11	M	37	104.0	Short	Conical
P12	M	58	104.3	Short	Conical
P13	M	17	88.9	Very short	Conical
P14	F	10	51.8	Short	Cylindrical
P15	F	67	77.6	Short	Conical
P16	M	12	107.0	Short	Conical
P17	F	9	64.5	Very short	Conical
P18	F	26	67.0	Short	Conical
P19	M	52	123.1	Short	Conical
**Average**		**30.2**	**76.3**		
**Std. Dev.**		**20.9**	**23.4**		

*Defined in accordance with Principles of Amputation, Physiopedia, as “Long”, “Short”, or “Very Short” [24].

**Defined in accordance with ISO 8548–3:2025 – Prosthetics and Orthotics — Limb Deficiencies — Part 3: Method of Describing the Residual Limb after Upper Limb Amputation, as “Conical”, “Cylindrical”, or “Bulbous” [25] .

Residual limbs for each participant were shape-captured by the treating prosthetist using standard plaster casting, then 3D scanned (Spectra Scanner, Vorum, Canada); the finished prosthetist-fabricated sockets were also scanned, yielding paired limb–socket meshes per participant. All prosthetists had over 5 years of experience in transradial socket design.

#### Data preparation.

**Fig 1:**
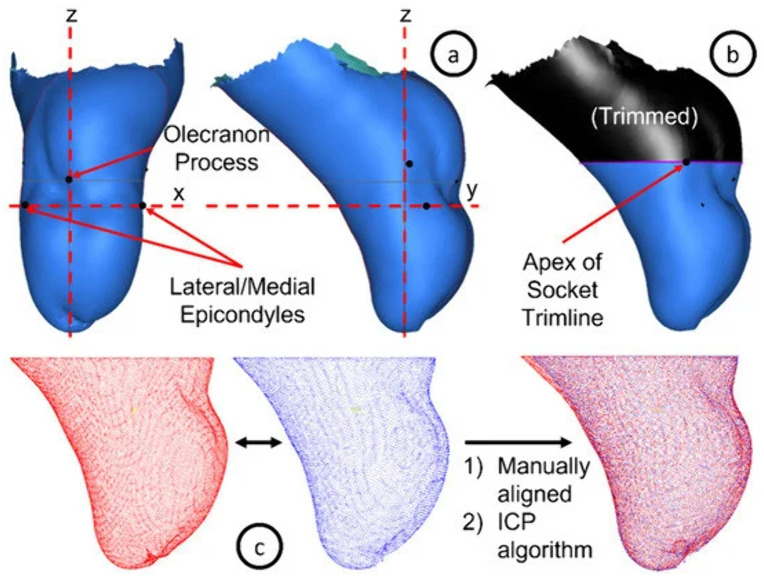
Coordinate convention used for aligning limb models (adapted from Ngan et al. [8]). The x-, y-, and z-Cartesian axes correspond to the frontal, sagittal, and longitudinal anatomical axes, respectively.

Post-processing of the limb–socket pairs was carried out using Canfit O&P and Spectra software (Vorum, Canada), which involved the alignment of all limb models using a previously established and validated anatomical landmark-based convention [8]. These landmarks, clearly marked on the limb by the treating prosthetist, included the medial and lateral epicondyles, as well as the olecranon process: bony prominences that remain unchanged and are readily identifiable. The x-, y-, and z-axes were aligned with the frontal, sagittal, and longitudinal axes, respectively (Fig 1). This alignment ensures that all subsequent shape comparisons conducted by the algorithm occur in corresponding anatomical regions and not arbitrary points in space.

**Fig 2:**
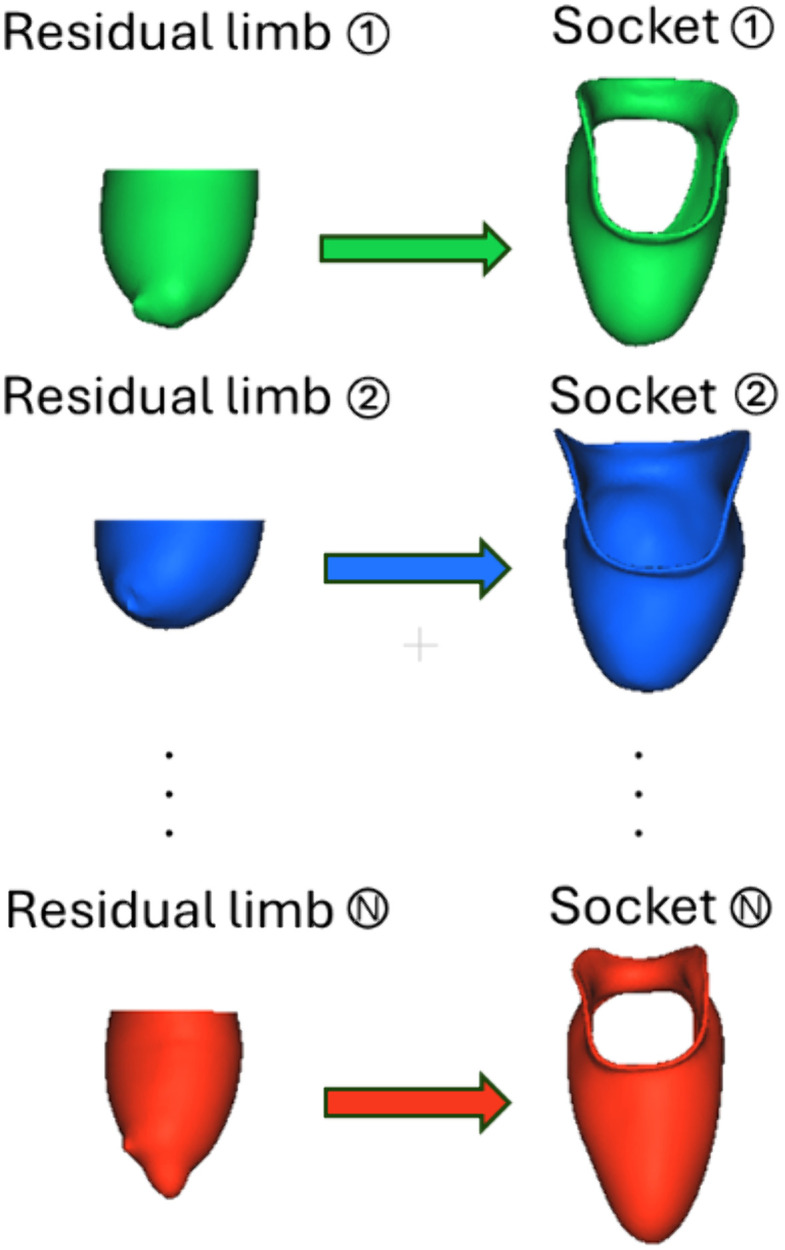
Reference library of limb–socket pairs (Obj 1a). This library is used by the algorithm to predict a socket based on the anatomical characteristics of a participant’s limb.

Similarly, each socket model was preliminarily aligned by setting the z-axis through its center and the distal tip. Finer registration between the socket and limb model was obtained by positioning the transverse plane perpendicular to the deepest indent in the proximal (superior) trimline (where the epicondyles would rest within the socket) and aligning the sagittal plane to pass through the olecranon tip or the olecranon obturator’s center. These registered limb–socket pairs for each individual participant formed the reference shape library used as the foundation for practice-driven transradial socket design (Fig 2), achieving **Obj 1a**.

#### Predictive algorithm for Customized Transradial Socket Design (PACT).

The development of the PACT is predicated on the fact that limbs with similar shapes often require similar socket designs [26,27]. While each limb is unique, a sufficiently large library of prosthetist-fabricated designs can account for a broad range of anatomical variations in residual limbs. In practice, there is no single “optimal” socket design, and a degree of variability is both expected and able to yield good patient outcomes, as demonstrated by intra- and inter-prosthetist differences observed in traditional socket fabrication [28,29]. By analyzing the shape of a new participant’s limb, the PACT predicts a custom transradial socket in three steps: 1) retrieve the most similar limb from the reference library, 2) select and isotropically scale the paired socket to the input limb’s dimensions, and 3) anisotropically scale in the A–P and M–L directions to improve localized fit.

**Fig 3:**
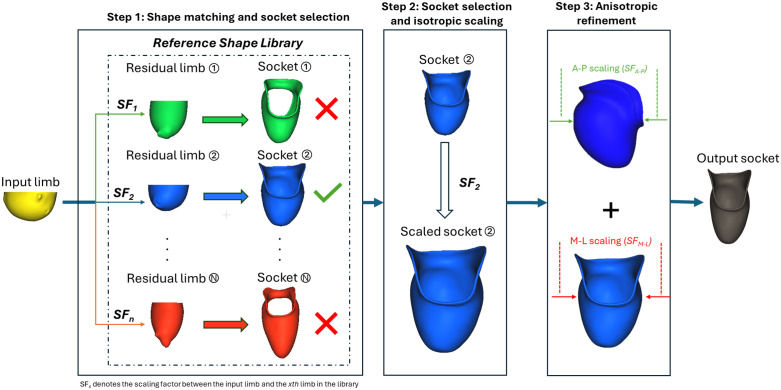
Flowchart of the algorithm. It comprises three main steps: shape retrieval, socket selection and isotropic scaling, and anisotropic refinement (Obj 1b).

These steps combine the limb’s quantifiable geometry with the clinician’s expertise embedded in the reference shape library through a “retrieve-and-refine” approach. While certain design elements in the library can be broadly applied across clients (such as trimline positioning and distal clearance), others (such as localized reliefs or functional preferences) are more case-specific and would require prosthetist intervention. The PACT geometrically scales and proportionally adjusts a retrieved socket to match the target limb’s dimensions, rather than reproducing all patient-specific modification steps. Detailed descriptions of each step are provided in the following sections, with a flowchart of the algorithm presented in Fig 3.

***Step 1—Shape retrieval*:** The first step compares the shape of the new limb to each limb in the reference library. Three main considerations ensure reliable comparisons:

1) **Alignment**: The new limb model is aligned to the standard reference frame using anatomical landmarks, as detailed in Sec 2.1.2, to ensure consistent comparison with the reference library in 3D space.
2) **Isotropic scaling**: To compare shape rather than size, all limb meshes are isotropically normalized based on their proximal–distal lengths, which is the primary mode of variation in limb shape [26,30,31]. For each library limb a unique scaling factor is applied:

$$\begin{array}{c} {SF}_{k}=\frac{L_{input}}{L_{k}} \end{array}$$ (1)

where *L*_*input*_ and *L*_*k*_ are the lengths of the input limb and the limb in the library, respectively.

3) **Shape-comparison metric**: After alignment and length normalization, similarity is measured as the average Euclidean (L2) distance between meshes P and Q sampled at n points:

$$d_{L\text{2}}(P,Q)=\sqrt{\frac{\text{1}}{n}\sum_{i=\text{1}}^{n}\|p_{i}-\;q_{i}\|{\;}^{\text{2}}}$$ (2)

where *p*_*i*_* *∈ *P* and *q*_*i*_ is its nearest point on *Q*. A lower *d*_*L2*_ indicates higher shape similarity, and this metric has proven effective for 3D shape retrieval [32,33].

***Step 2—Socket selection*:** Using the L2-distance from **Step 1**, the PACT automatically selects the closest-matching limb in the library and retrieves its paired socket in **Step 2**. Previous research has shown that there are general trends in the design approach for transradial prosthetic sockets [9]. For example, while a child’s limb and an adult’s limb differ in absolute dimensions, if their relative anatomical proportions are similar when scaled to the same proximal–distal length, then the underlying socket design principles should remain applicable. Therefore, since every limb in the library was already resized to the client’s limb length in **Step 1**, we apply the same uniform scaling factor (*SF*_*k*_) to the selected socket model. This approach allows the socket to conform to the key anatomical contours necessary for optimized fit and load distribution, producing a first-pass socket that matches the new limb’s overall dimensions [34–36].

**Fig 4:**
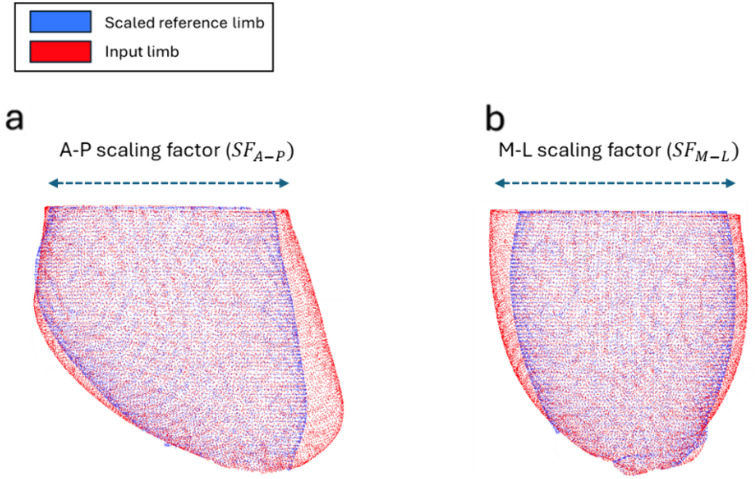
Calculating anisotropic scaling factors. **a)** Calculating SF_A–P_ (sagittal view); **b)** Calculating SF_M-L_ (frontal view).

***Step 3—Anisotropic refinement*:** The template socket obtained in **Step 2** is then further customized to ensure optimal fit for the input limb in both the principal cross-sectional directions by calculating additional anisotropic scaling factors (illustrated in Fig 4):

- **Anterior–posterior scaling factor, *SF***_***A-P***_.

Defined, in the same manner as Eq. 1, as


$$\begin{array}{c} {SF}_{A-P}=\frac{A-P\;dimension\;of\;input\;limb}{A-P\;dimension\;of\;the\;scaled,\;matched\;limb} \end{array}$$ (3)

- **Medio-lateral scaling factor, *SF***_***M-L***_.

Analogously,


$$\begin{array}{c} {SF}_{M-L}=\frac{M-L\;dimension\;of\;input\;limb}{M-L\;dimension\;of\;the\;scaled,\;matched\;limb} \end{array}$$ (4)

Both factors are applied to the template, yielding an anisotropically scaled socket design that globally encompasses the input limb while preserving other geometric features inherited from the socket retrieved from the reference library.

#### Implementation details.

All processing was implemented in Python 3.8.16 (Anaconda 2023.09). Key packages and versions were as follows: Numpy 1.23.5, Scipy 1.10.1, scikit-learn 1.2.2, trimesh 4.0.3 (mesh sampling, voxelization), and Meshlab 2020.07 (ICP and L2-distance computation). End-to-end runtime averaged 13.2 ± 0.7 s per limb when run serially on typical modern hardware.

### Global and local algorithm evaluation*—*Obj 2

#### Global validation of PACT-predicted sockets.

We compared the overall similarity of PACT-predicted sockets to their prosthetist-fabricated counterparts (the gold standard) in terms of the surface deviations and volumes. We also evaluated the cross-sectional area (CSA) variations along the model using a novel slice-based approach to identify where along the length of the socket the predictions differed the most from prosthetist designs.

***Overall prediction accuracy*:** This analysis evaluated the overall accuracy of the final socket predictions produced by the PACT. For each of the 19 residual limbs in the reference library, the corresponding prosthetist-fabricated socket was temporarily excluded from the library, and a socket was predicted using the remaining data. This predicted socket was then compared to the gold-standard prosthetist-fabricated socket for the participant by converting the sockets into point clouds, performing rigid co-registration, and finally assessing similarity using the 1) Euclidean (L2) distances and 2) volumes. The goal was to determine whether the PACT-predicted sockets closely approximate those designed by prosthetists, thereby demonstrating their potential for clinical application.

**Fig 5:**
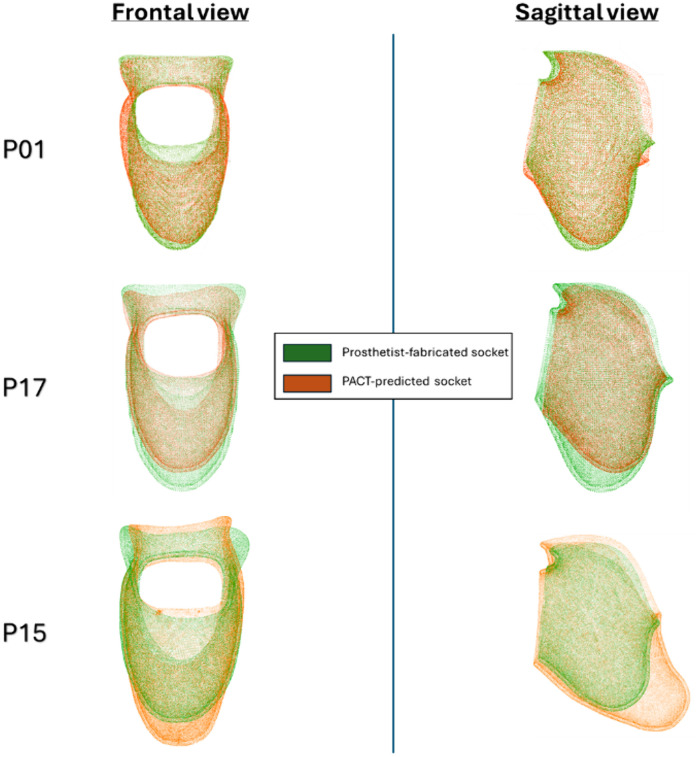
Aligned point clouds of the predicted and prosthetist-fabricated sockets for P01, P17 (youngest participant), and P15 (oldest participant). Note that P15 represents one of the worst predictions: explanations for this are provided in the discussion.

The point clouds were preliminarily aligned using centroid alignment, and refined using the iterative closest points (ICP) algorithm. Fig 5 shows the aligned point clouds for three participants: P01 (median error), P17 (youngest participant), and P15 (oldest participant).

Following alignment, the similarity between each pair of sockets was quantified by 1) pairing each point with its nearest neighbor in the corresponding point cloud and computing the L2-distance between them. Next, the 2) percentage difference in volumes of the two point clouds was compared using the following formula:


$$\begin{array}{c} V_{diff}=\text{1}\text{0}\text{0}\times \frac{V_{PFS}\;-\;V_{PACT}}{V_{PFS}} \end{array}$$ (5)

where *V*_*diff*_ is the percentage difference between sockets, *V*_*PFS*_ is the volume of the prosthetist-fabricated socket, and *V*_*PACT*_ is the volume of the PACT-predicted socket.

**Fig 6:**
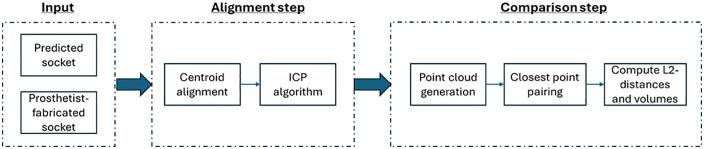
Flowchart of the alignment and comparison process for validating the accuracy of the PACT.

The average L2-distances and volume differences between predicted and prosthetist-fabricated sockets across the dataset can indicate whether the algorithm is generating reasonable approximations of prosthetists’ designs (i.e., within 3 mm surface deviation and 5% volumetric difference, thresholds that are commonly cited to be ‘good fits’ in clinical practice [28,29,37]). A flowchart of this validation process is shown in Fig 6.

**Fig 7:**
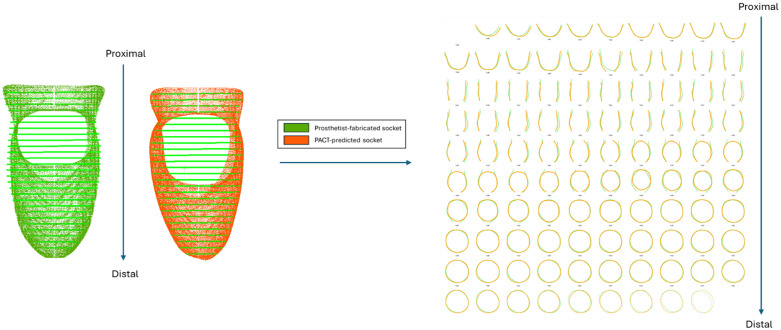
Slicing along the proximal–distal axis for both the prosthetist-fabricated and PACT-predicted sockets (P01).

***Variation in cross-sectional area*:** To examine how well the PACT preserves the geometry along the entire length of the socket, we first sliced both the prosthetist-fabricated socket and the PACT-predicted socket along the proximal–distal axis at 100 equal intervals, from 0% (distal tip) to 100% (top of the proximal trimline). An example of this slicing process is shown in Fig 7, for P01, where the slices for the prosthetist-fabricated and PACT-predicted socket have been overlaid.

After slicing both sockets, the percentage differences in the cross-sectional area for each slice *j* of participant *k* using Eq. 6:


$$\begin{array}{c} \Delta_{k,j}=\frac{A_{k,j}^{PACT}\;-\;A_{k,j}^{PFS}\;}{A_{k,j}^{PFS}}\times \text{1}\text{0}\text{0} \end{array}$$ (6)

where the superscripts *PACT* and *PFS* denote PACT-predicted and prosthetist-fabricated sockets, respectively. Using this formula, slice-wise averages and standard deviations of the cross-sectional area were calculated for all participant pairs.

#### Identifying localized socket deviations.

While global comparisons can assess the overall quality of the predictions, it is imperative to also identify specific regions where the predicted sockets consistently deviate from their prosthetist-fabricated counterparts. Identifying these localized differences can facilitate the targeted improvement of the PACT through the incorporation of local mesh modifications.

**Fig 8:**
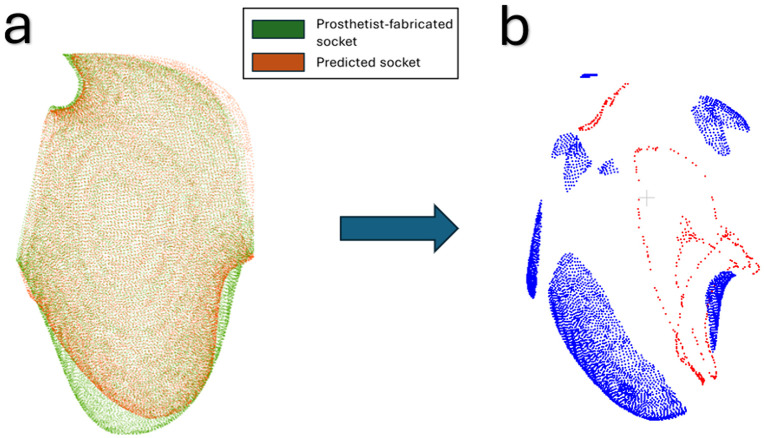
Signed-distance color map generation for P01. **(a)** aligned prosthetist-fabricated and PACT-predicted sockets; **(b)** color map of pronounced deviations between the two sockets (red signifies where the predicted socket was larger, and blue is where it was smaller).

***Signed-distance color map generation*:** For each predicted socket, the signed surface distances to its prosthetist-fabricated counterpart were calculated, where positive values indicate that the predicted socket is larger and negative values indicate where the predicted socket is smaller. Following a previous approach [9], pronounced deviations were then flagged through establishing thresholds by separating the positive and negative signed distances into two groups, where the mean value for each group acted as its respective threshold. These deviations are visualized as a color map in Fig 8.

***Common deviations across participants*:** Once these color maps were generated, the next step was to isolate the most pronounced deviations using machine learning. We applied the DBSCAN algorithm to the areas marked as pronounced deviations, yielding distinct clusters where predictions differ substantially from prosthetist-fabricated sockets and improving the interpretability of the color maps [38,39].

To find recurring regions across participants, we (i) normalized clusters to a common scale, (ii) computed each cluster’s centroid, (iii) aggregated centroids from all participants into a sparse point cloud, and (iv) re-applied DBSCAN. Finally, we expressed each region’s location as an average unit direction vector from the anatomical origin, which abstracts scale and radial distance while preserving angular position [9].

#### Subgroup statistical analysis.

To further understand whether participant characteristics (listed in Table 1) influence PACT performance, their effects on the two variables affecting global accuracy (volume differences and mean L2-distances) were analyzed. Four binary categorical variables (characteristics) were considered: sex (male/female), limb shape (conical/cylindrical), limb length (short/very short), and age group (pediatric <18 y/adult ≥18 y). For each binary factor, group means and standard deviations were calculated and compared using the Welch two-sample t-test, which does not assume equal group variances and works well for small groups with an uneven number of samples [40].

Further, to increase statistical power, we assessed age as a continuous variable instead of simply a binary one (i.e., pediatric vs non-pediatric subgroups) using the Pearson’s *r*, which measures the strength and direction of a linear relationship between two continuous variables [41]. Statistical significance was set at p < 0.05 (two-tailed), because differences in the mean in either direction were of interest. The continuous-age analyses were treated as primary for age-related effects, while the pediatric/adult comparison was retained for clinical interpretability.

Given the modest sample size (n = 19) and the number of subgroup comparisons, these analyses were considered exploratory. We report uncorrected p-values in Table 5 for transparency, and additionally applied a Holm–Bonferroni correction across all subgroup tests (four binary factors × two outcomes plus two age correlations; 10 tests total).

**Table 5:** Subgroup outcomes for mean volume differences and L2-distances.

Factor	Subgroup (n)	Mean volume difference ± SD (%)		Mean L2-distance ± SD (mm)	
Sex	Female (9)	–1.0 ± 3.7	*p* = 0.43	1.91 ± 0.56	**p = 0.034**
	Male (10)	+0.4 ± 4.0		2.49 ± 0.53	
Limb shape	Conical (10)	+0.58 ± 4.12	*p *= 0.34	2.42 ± 0.66	p = 0.12
	Cylindrical (9)	–1.12 ± 3.42		1.99 ± 0.47	
Limb length	Short (12)	–0.70 ± 3.22	p = 0.54	2.22 ± 0.64	p = 0.93
	Very short (7)	+0.59 ± 4.81		2.20 ± 0.58	
Age	Pediatric (9)	–2.12 ± 3.10	**p = 0.033**	2.05 ± 0.53	p = 0.27
	Adult (10)	+1.48 ± 3.68		2.36 ± 0.65	

## Results

As outlined in Sec 2.1.3, the PACT applies one global (isotropic) and two directional (anisotropic) scaling coefficients to each retrieved socket. Table 2 reports these coefficients for all 19 leave-one-out predictions: isotropic scaling factor *SF*_*k*_, along with the anisotropic medio-lateral and anterior–posterior scaling factors *SF*_*M–L*_ and *SF*_*A–P*_, respectively. Across the dataset *SF*_*k*_ = 1.00 ± 0.23 (0.64–1.55), *SF*_*M–L *_= 0.99 ± 0.07 (0.89–1.13), and *SF*_*A–P*_ = 0.99 ± 0.11 (0.80–1.26).

**Table 2:** Scaling factors applied by PACT for each participant.

Scaling factor	P01	P02	P03	P04	P05	P06	P07	P08	P09	P10	P11	P12	P13	P14	P15	P16	P17	P18	P19	Range (Min-Max)
*SF*_*k*_ (Isotropic)	0.75	0.75	0.77	0.99	1.01	1.17	1.14	1.01	1.36	0.82	1.55	0.97	1.33	0.87	0.99	1.03	0.83	0.64	1.15	**0.64–1.55**
*SF*_*M–L*_ (Anisotropic)	0.89	0.98	1.02	0.97	1.04	1.05	1.01	1.13	1.07	0.92	1.00	1.00	1.12	0.99	0.89	1.00	0.96	1.00	0.93	**0.89–1.13**
*SF*_*A–P*_ (Anisotropic)	1.02	0.91	1.07	1.11	0.90	0.94	1.11	0.97	0.89	1.10	1.06	1.26	0.98	0.90	1.04	0.80	0.91	0.94	0.98	**0.80–1.26**

### Global validation of PACT-predicted sockets

#### Overall prediction accuracy.

**Fig 9:**
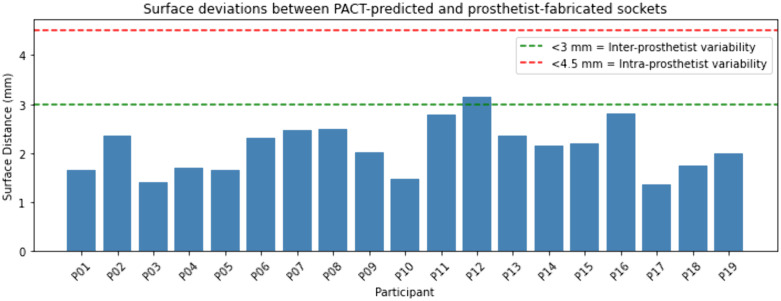
Mean Euclidean (L2) distance between PACT-predicted and prosthetist-fabricated socket for each participant. The green dashed line (3 mm) denotes the reported upper limit of *inter-prosthetist* surface-deviation, while the red dashed line (4.5 mm) marks the worst-case *intra-prosthetist* variability found in lower-limb socket applications.

The overall prediction accuracy was verified using the average L2-distances and volume differences between the PACT-predicted and prosthetist-fabricated sockets. Across all participants (n = 19), the average absolute Euclidean (L2) distance was found to be 2.11 ± 0.51 mm. Fig 9 shows the mean L2-distances for each participant; it can be seen that the maximum and minimum deviations are 3.16 mm and 1.36 mm, respectively. Two reference bands are superimposed on the figure to represent inter-prosthetist [29] and worst-case intra-prosthetist variability [28] in lower-limb applications, which provides clinical context.

**Fig 10:**
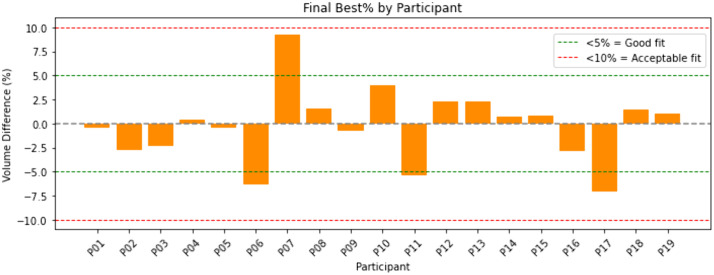
Signed volume differences (%) between PACT-predicted and prosthetist-fabricated socket for each participant. The green dashed band (± 5%) marks the “good-fit” range reported by Fernie and Holliday [37], while the red dashed band (± 10%) indicates the “acceptable-fit” range.

Next, the volume differences between PACT-predicted and prosthetist-fabricated sockets were calculated for each participant using Eq. 5. The average absolute difference was found to be 2.74 ± 2.56%, with maximum and minimum absolute values of 9.3% and 0.4%, respectively. Fig 10 shows the signed volumetric differences, where a negative sign indicates that the PACT-predicted socket is smaller than its prosthetist-fabricated counterpart, while a positive sign indicates the opposite. Again, four reference bands are superimposed on the figure to represent “good fit” and “acceptable fit” criteria proposed in literature [37].

#### Variation in cross-sectional area.

**Fig 11:**
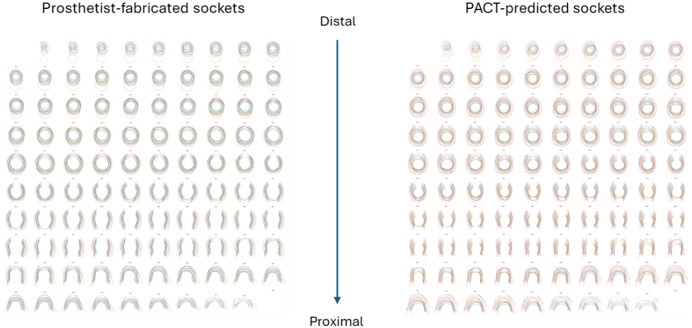
Superimposed cross‑sectional contours of prosthetist‑fabricated (left) and PACT-predicted sockets. Plotted at 100 equally spaced positions from the distal tip to the proximal trimline.

PACT-predicted sockets closely reproduced the geometry of prosthetist-fabricated sockets along the proximal–distal length. A composite overlay of the slices for all participants is shown in Fig 11 to verify the slice-wise agreement.

**Fig 12:**
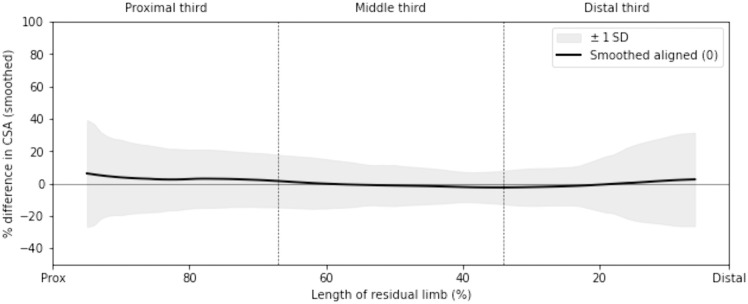
Mean ± 1 SD of the percentage difference in cross-sectional area (CSA) between PACT-predicted and prosthetist-fabricated sockets for 100 slices along the socket length. Values are averaged across all participants, with 100% corresponding to the proximal trimline and 0% to the distal tip.

For every slice, the cross-sectional areas of the PACT-predicted and prosthetist-fabricated sockets were computed, and their percentage difference was determined using Eq. 6. This slice-wise percentage difference was calculated for every participant, and the averaged profile across participants is plotted in Fig 12. Overall, the mean difference is generally below 10% across the socket length, and a trend can be observed: larger differences are found at the proximal and distal thirds, while the middle third shows comparatively better agreement between the predicted and prosthetist-fabricated sockets.

### Identifying localized socket deviations

The goal of this section is to determine which regions on the predicted sockets consistently diverge from prosthetist-fabricated sockets through cluster analysis.

#### Signed-distance color map visualization and cluster analysis.

**Fig 13:**
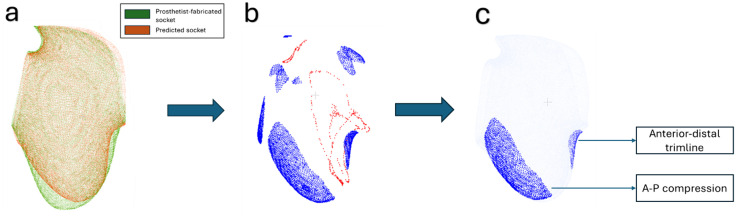
Generating clusters for P01 using DBSCAN. **(a)** Aligned predicted and prosthetist-fabricated sockets. **(b)** Signed-distance color maps illustrating localized differences in socket geometry. **(c)** Clusters identified by DBSCAN for P01— the anterior-distal trimline and anterior–posterior compression regions.

Fig 13 shows representative signed-distance color maps and DBSCAN clusters for P01, highlighting localized differences between predicted and prosthetist-fabricated sockets. Distinct regions of deviation were found for all participants, which are summarized in Table 3. Out of the 19 participants, 15, 11, 5, and 4 participants showed deviations in the anterior-distal trimline, A–P compression, distal tip, and supracondylar compression regions, respectively. These findings indicate that, while the PACT captures the overall geometry effectively, certain socket regions require further refinement.

**Table 3:** Presence of localized socket deviations across participants, summarized by region.

	P01	P02	P03	P04	P05	P06	P07	P08	P09	P10	P11	P12	P13	P14	P15	P16	P17	P18	P19
**Anterior-distal trimline**	✓	✓	✓			✓	✓	✓		✓	✓		✓	✓	✓	✓	✓	✓	✓
**AP-compression**	✓			✓	✓	✓	✓		✓	✓	✓	✓						✓	✓
**Distal tip**		✓	✓					✓		✓							✓		
**Supracondylar compression**				✓	✓		✓		✓										

#### Common deviations across participants.

**Fig 14:**
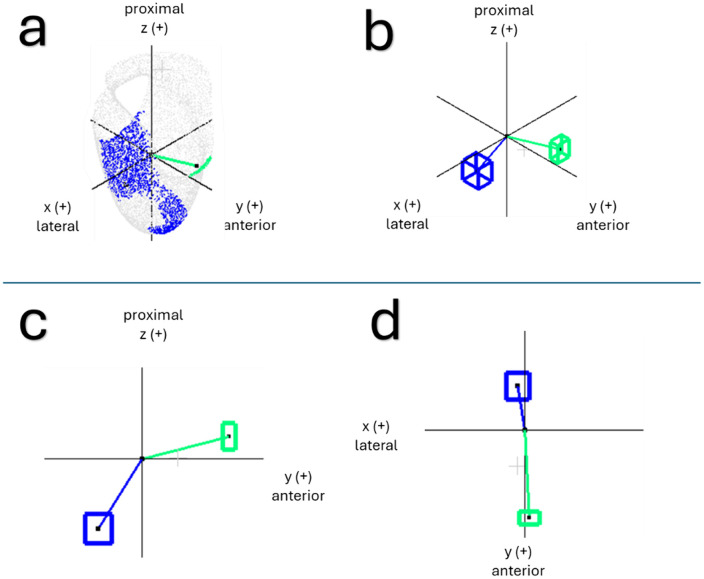
Average unit direction vectors for the two primary regions of deviation. **(a)** example of 3D vectors from the origin to the centroid of each region of deviation in the prosthetist-fabricated socket; **(b)** average vectors presented in a unit circle, where the boxes represent one standard deviation in each cartesian direction; **(c)** unit vectors viewed in the y–z plane; and **(d)** unit vectors viewed in the x–y plane.

A second DBSCAN analysis was conducted on the centroids of the previously identified clusters for all participants. In accordance with Table 3, DBSCAN found that the most common deviations across the dataset were at the anterior-distal trimline and anterior–posterior compression (A–P compression) regions. Table 4 and Fig 14 summarize the average unit direction vectors of these regions of deviation across participants.

**Table 4:** Average unit vectors of the two main regions of deviation.

Region of deviation	Average unit direction vector
Anterior-distal trimline	$-\text{0}.\text{0}\text{5}\;(\text{0}.\text{2}\text{2})\;\hat{x}\;+\;\text{0}.\text{9}\text{7}\;(\text{0}.\text{1}\text{4}\hat{y\;}+\;\text{0}.\text{2}\text{5}\;(\text{0}.\text{2}\text{9})\;\hat{z}$
A–P compression	$-\text{0}.\text{0}\text{9}\;(\text{0}.\text{2}\text{8})\;\hat{x}\;-\;\text{0}.\text{5}\text{3}\;(\text{0}.\text{3}\text{3}\hat{y}\;-\;\text{0}.\text{8}\text{4}\;(\text{0}.\text{3}\text{6})\;\hat{z}$

Values in parentheses indicate one standard deviation.

### Subgroup analysis

**Fig 15:**
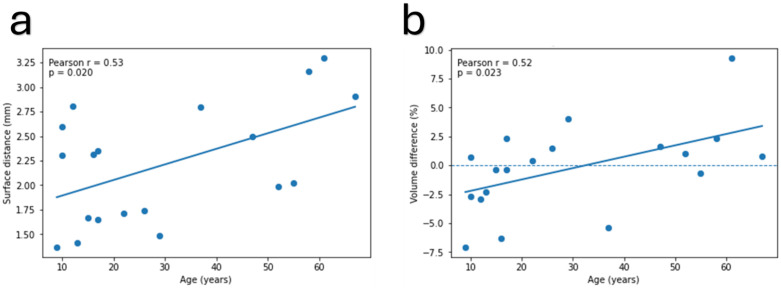
Relationship between age and PACT performance metrics. Both **(a)** mean L2-distance (r = 0.53, p = 0.020) and **(b)** mean volume difference (r = 0.52, p = 0.023) show moderate positive correlations, indicating errors increase with age.

Table 5 reports the mean ± SD values for the mean volume differences and Euclidean (L2) distances of all participant subgroups, alongside the Welch two-sample *p*-values. In these uncorrected analyses, we observed lower mean L2-distances for female versus male residual limbs (p = 0.034) and more negative volume differences for pediatric versus adult participants (p = 0.033). When age was treated as a continuous variable, moderate positive correlations were observed between age and both mean volume difference (r = 0.52, uncorrected p = 0.023) and mean Euclidean (L2) distance (r = 0.53, uncorrected p = 0.020), suggesting that errors tended to increase with age, as shown in Fig 15.

However, these subgroup analyses involved 10 statistical tests in total (four binary factors × two outcomes plus two age correlations). When we applied a Holm–Bonferroni correction across all tests, none of the subgroup effects remained statistically significant at α = 0.05 (Holm-adjusted p-values ranged from approximately 0.20 to 1.00). Therefore, the patterns reported in Table 5 should be interpreted as exploratory rather than definitive evidence of systematic differences in PACT performance across subgroups.

## Discussion

Towards the development of a prosthetist practice-driven socket design, this study presents the first automated “retrieve-and-refine” pipeline capable of generating transradial sockets from a single limb scan in minutes. In 19 participants, the PACT produced sockets with an average surface deviation of 2.11 ± 0.51 mm from prosthetist-fabricated sockets—comparable to intra- and inter-prosthetist variability [28,29]—and mean volumetric differences of 2.74 ± 2.56%, within the 5% “good fit” threshold for lower-limb sockets [37]. These results indicate that, with further refinements and a larger library, the PACT could offer a clinically viable method for producing usable first-draft socket designs.

### Global validation of PACT-predicted sockets

#### Overall prediction accuracy.

In this study, across all participants (n = 19), the Euclidean (L2) distances between PACT-predicted sockets and their corresponding prosthetist-fabricated sockets were found to be 1.36–3.16 mm. While no studies specific to transradial sockets exist, previous research has found that the L2-distances between two manually casted transtibial sockets by the same prosthetist are 2.79–4.41 mm [28]. Given these values reflect a single prosthetist’s casts, inter-prosthetist differences, which potentially involve varied techniques, would likely be even greater. Further, another study found that, in zones of major rectification, the inter-prosthetist variability was 1–3 mm for patellar-tendon-bearing sockets [29]. This value likely underestimates the overall variability because prosthetists pay special attention to the major rectification zones, ensuring that modifications in these critical areas are very consistent, which results in lower measured variations. Our localized deviations (1.36–3.16 mm; Fig 9) are within these ranges; however, it is important to note that these benchmark values are for lower-limb sockets, which are generally larger than upper-limb ones. Nevertheless, literature has demonstrated that intra- and inter-prosthetist variability on the order of 1–4 mm in surface shape is common [28,29], indicating that discrepancies of this magnitude between the PACT’s design and a prosthetist-made socket are within clinically acceptable limits.

Another key metric affecting user comfort is the volume of the socket [21,42]. Previous research has found that a 0–5% difference in socket volume is “good” fit, a 5–10% difference is “acceptable” fit, while a difference of 10% or more indicates the need for a new socket [37]. According to this criterion, 15 of the PACT-predicted sockets qualify as “good” fit and the remaining 4 as “acceptable” fit, with none requiring replacement (Fig 10). Sanders later reported that a 6% volume mismatch is where a decline in fit begins to be observed [42]. Using this cut-off, 16 PACT-predicted sockets had good fit, while 3 did not. While, again, it is important to note that both of these studies focused on the lower limb; analyses using percentages do allow for a size-adjusted comparison of upper-limb.

The volumetric comparisons show that most participants’ predicted sockets fall within the “good-fit” range, but three clear outliers exist: P06, P07, and P17. For these cases, the algorithm retrieved limbs P07, P17, and P15 as the closest matches, and the repeated appearance of P07 and P17 suggests that these two limb shapes (“very short”/ “conical”) are under-represented in the 19-participant library. Age and soft-tissue compliance may also play a role: P07 is the second-oldest participant, while P17 is the youngest. Furthermore, from Fig 5, it can be seen that the predicted socket for P15 deviates drastically from the prosthetist-fabricated one at the distal end. This error likely propagated through the scaling process as it was based on the socket for P08, who had a particularly elongated distal end. These factors highlight the need for a larger and more diverse reference set, particularly “long“/ “bulbous” and “very short”/ “conical” limb types (Table 1), in addition to tagging or down-weighting special case designs (such as the one for P08) so clinicians are alerted when a predicted socket reflects outlier geometry. Further, future versions of PACT should incorporate tissue-stiffness and age-related factors, which can be used to customize regions such as the distal tip. Because transradial users form the largest upper-limb amputee group, multi-center collaborations and routine digital workflows would greatly accelerate the collection of such data and improve retrieval accuracy [5,6,12].

Evidence from related work supports this direction. Data-driven shape-library studies typically enroll 20–30 participants, which is slightly above the range in this study [14–17]. While these studies prove technical feasibility, they are by no means an optimal target. Further, in lower-limb applications, researchers have addressed the limited availability of objective data by proposing statistical shape models that describe residual limb variations and define worst-case sizes and shapes [43]. For instance, Dickinson et al. [26] found that 40 lower-limb training models could capture the essential modes of variation in lower limbs with a mean accuracy of 1 mm, while Khan et al. [44] used 51 femur models to capture the overall size and shape differences in anatomy. Another study used 35 lower-limb models to reliably reconstruct bone surfaces with an error of about 2 mm [45]. These findings indicate that while a sample size between 30 and 50 is sufficient to capture anatomical variability across the lower-limb population, larger numbers are likely to improve the results for upper-limb applications, which differ in terms of anatomy, soft-tissue composition, and socket design requirements. Adopting similar methods for upper-limb applications can help to fully understand the variations in anatomy across the population, facilitating the creation of a more comprehensive reference shape library. This would, in turn, enhance the performance of the PACT by ensuring that it can account for a broader range of anatomies.

#### Variation in cross-sectional area.

The composite overlay in Fig 11 highlights the visual similarity between predicted and prosthetist-fabricated sockets sampled at the same 1% increments. To quantify this observation, the percentage difference in cross-sectional area was computed slice-by-slice and averaged across all 19 participants (Fig 12). The slice-wise mean CSA error stays below ±10% for almost the entire socket length, matching the “acceptable” fit band proposed by Fernie and Holliday [37]. It is also evident that the CSA differences peak at the distal and proximal ends. Recent shape-analysis work on transradial socket rectification reports the same trend, where both ends of the rectified socket model exhibited higher CSA differences [9].

- **Proximal third:** The errors peak at the proximal end and quickly reduce towards the middle of the socket. Prosthetists frequently add a patient-specific trimline in this region, so a purely geometry-based algorithm will tend to mispredict the cross-sectional area here.
- **Middle third:** The mean cross-sectional area hovers around 0% here, and the narrow ±1 SD envelope indicates that the load-bearing portion of the socket is modeled consistently.
- **Distal third:** A second rise in error occurs where the socket cups the distal end of the limb. This could be because the cross-sectional areas of slices in these regions are small; even small radial discrepancies could inflate the percentage difference. Further, prosthetists often leave reliefs at the distal end – these are quite subject-specific and have been found to be the most inconsistently modeled region of the socket [9].

While these global CSA variations provide insight into which regions of the socket are modeled better by the PACT, a more localized analysis was conducted to identify the specific regions on the socket that were consistently mispredicted by the PACT.

### Localized socket deviations

**Fig 16:**
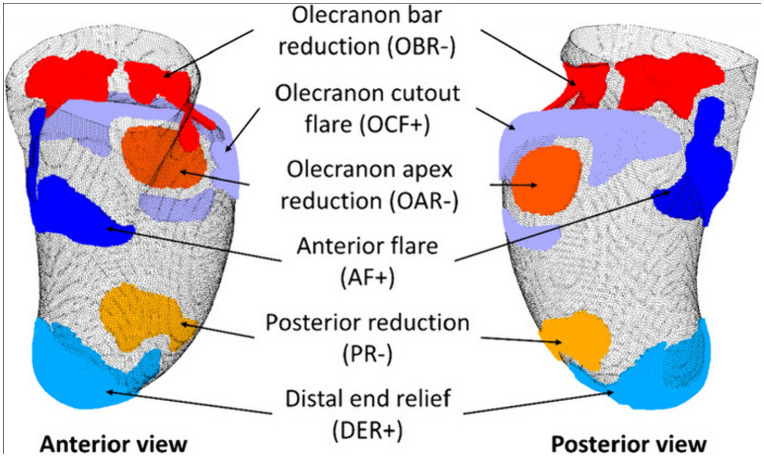
Localized rectification zones identified in a study by Ngan et al. [9].

To interpret the localized shape differences in a clinically relevant manner, we compare our findings to the recent study by Ngan et al. [9], which quantified rectification for the three-quarter Northwestern-style transradial socket and identified six common rectification zones where prosthetists add or remove material (Fig 16). These are: (1) olecranon bar reduction (OBR): posterior–proximal reduction superior to the olecranon to load the triceps tendon and contribute to suspension/stability; (2) posterior reduction (PR): posterior–distal reduction that increases anterior–posterior (AP) compression for an intimate fit; (3) anterior flare (AF): addition along the anterior trimline to prevent tissue impingement and facilitate donning/doffing; (4) olecranon cutout flare (OCF): addition that forms the posterior obturator flare around the olecranon to improve ventilation and elbow range of motion; (5) olecranon apex reduction (OAR): posterior–proximal reduction at the olecranon apex as part of the three-quarter opening; and (6) distal end relief (DER): addition at the distal end to reduce pressure and, in pediatrics, accommodate growth.

**Fig 17:**
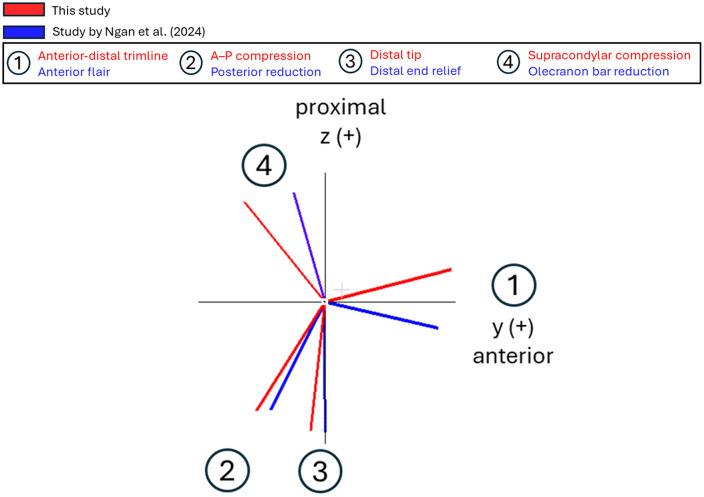
Correspondences between the regions of deviation in this study and rectification zones identified by Ngan et al. [9]. Unit vectors for all regions of deviation viewed in the y–z plane. Note: The spreads in the other two planes are too low to visualize meaningfully.

In this study, Fig 13 and Table 3 show that recurrent PACT deviations concentrate at (i) the anterior-distal trimline (15/19) and (ii) A–P compression (11/19). Less frequent clusters appeared at the distal tip (5/19) and the supracondylar compression region (4/19). To verify spatial consistency, we compared the average unit direction vectors of all our regions of deviation to the vectors reported by Ngan et al. [9]; AF corresponded closely to our anterior-distal trimline cluster, PR to our AP-compression cluster, DER to our distal tip cluster, and OBR to our supracondylar compression cluster (Fig 17).

Below, we discuss each region, in descending order of incidence:

#### Anterior flare (AF).

The most frequently occurring deviation (15/19) in this study was the anterior-distal trimline, which facilitates weight-bearing and the allowance of tissue bunching during flexion. Socket guidelines state that the distance between the anterior-distal trimline and the distal end of the socket must exceed 45% of the length of the limb [19]. In this study, all PACT-predicted sockets met this condition, with an average distance of 61.34 mm for limbs averaging 76.3 mm in length (approximately 81%). However, localized deviations in this region suggest that further refinement could enhance the balance between limb entrance and distal clearance. This could be mitigated by increasing the size of the reference library to include additional trimline types, or by allowing the prosthetist to digitally modify the trimlines prior to client fittings.

#### Posterior reduction (PR).

The second most frequent deviation (11/19) found in this study was anterior–posterior compression, which provides stability and improved load distribution [9]. This compression is significantly dependent on compressing soft tissue to improve stability: currently, the PACT does not account for soft-tissue characteristics. Future versions of the algorithm could integrate clinician-inputted values, either through qualitative assessments or quantitative tools such as indentometers [46], that directly capture the desired A–P compression value. Incorporating such measurements can facilitate individualized socket design that considers users’ soft-tissue properties. In the present study, soft-tissue amount and compliance were assessed qualitatively during clinical care but were not recorded in a standardized or quantitative form.

#### Distal end relief (DER).

A less frequent deviation (5/19) was found at the distal tip of the PACT-predicted sockets, a zone that reduces pressure, accommodates tissue, and allows for pediatric growth. The subgroup analysis in Sec 3.3 clearly shows why: the PACT takes no age-related factors into account. Therefore, overall, the PACT-predicted sockets are larger than their prosthetist-fabricated counterparts for older participants and smaller for pediatric participants, where prosthetists leave additional room for growth. The incorporation of metadata (such as age and soft-tissue characteristics) can help customize this region in terms of fit and function.

#### Olecranon bar reduction (OBR).

The least frequent observed deviation (4/19) was at the supracondylar compression region, which provides suspension and stability. Table 2 can help explain why: the limb length varies widely, so the isotropic length-based scaling factor *SF*_*k*_ spans 0.64–1.55 (range = 0.91). However, the anisotropic scaling factors *SF*_*M–L*_ and *SF*_*A–P*_ are much tighter: 0.89–1.13 (range = 0.24) and 0.80–1.26 (range = 0.46), respectively. The range for the medio-lateral scaling factor is nearly half that of the anterior–posterior scaling factor, indicating that the library contains limbs with similar medio-lateral regions to the input scans, necessitating only minor corrections in this region; this explains the good performance in the supracondylar region. However, incorporating prosthetist-measured values into the algorithm can help reach millimetre-accurate shaping in this region, improving socket suspension.

#### Olecranon cutout flare (OCF) and Olecranon apex reduction (OAR).

These two regions are involved in shaping the posterior obturator in the olecranon region to improve ventilation and range of motion. As such, these regions did not appear as distinct, recurring clusters of deviation in PACT-predicted sockets. This implies that the PACT generally models these regions well; however, improvements can be made if the width and height of the obturator can be parameterized and explicitly tuned, rather than being inherited directly from the library.

Overall, these patterns of misprediction highlight specific regions where the PACT does not consistently replicate prosthetist-made designs. Identification of these recurring patterns indicate the need for clinician-led modifications or further localized adjustments to improve socket fit. These enhancements could improve the accuracy of patient-specific socket designs by addressing aspects of socket geometry that are not fully captured by the current predictive algorithm.

### Subgroup analysis

Subgroup analysis (Table 5) found two notable trends:

- **Age**: On average, the PACT predicted slightly undersized sockets for pediatric users and slightly oversized ones for adults. Continuous-age correlations also showed that both volume error and surface distance error rise steadily with age (Fig 15). This is corroborated by the edge cases shown in Fig 5. There are two potential clinical factors that drive this trend. Firstly, clinicians routinely add clearance for children, who may gain limb volume due to growth between visits. Secondly, older users often have stiffer and less compressible soft tissue; therefore, they receive closer-fitting sockets without leaving room for growth. Since the PACT currently relies on length-based scaling, it does not account for these age-related adjustments; incorporating tissue-stiffness measurements or age-specific socket matching could reduce this bias.
- **Sex**: Female residual limbs showed an approximately 0.6 mm lower mean surface deviation than their male counterparts (*p* = 0.034). Several explanations are plausible. First, as shown in Table 1, the female subgroup in this dataset had a lower mean age (23y; SD = 18y) than the male subgroup (37y; SD = 21y) indicating a smaller overall limb volume. This is further confirmed by the average residual limb length for the female subgroup being 63.36 mm (SD = 16.6 mm), which is smaller than that of the male subgroup, at 87.8 mm (SD = 23.2 mm). Sockets for larger limbs will naturally show larger errors in magnitude. Second, the sample is small (n = 9 vs 10) and the variance in female L2-distance is already lower; a single additional female outlier could remove the significance. Larger, more balanced cohorts are needed before drawing any firm clinical conclusions.

No significant effects were found for distal-tip morphology or residual-limb length, indicating that the existing length-normalisation and anisotropic refinement steps manage those anatomical variations adequately within the present dataset. However, long or bulbous limbs are still absent from the reference library; adding such shapes will provide a stricter test of this conclusion.

Overall, the subgroup analysis highlights two priorities for future development: (1) augment the scaling strategy with soft-tissue compliance or age-specific rules such that pediatric and adult users receive equally accurate first-pass designs and (2) expand the reference library with a wider age range and rarer limb morphologies.

These subgroup findings should be interpreted with caution. The analyses involved 10 statistical tests on a relatively small sample, and although some effects reached p < 0.05 in the uncorrected analyses, none remained statistically significant after Holm–Bonferroni correction (smallest Holm-adjusted p ≈ 0.20). These age- and sex-related patterns should be treated as exploratory trends that highlight priorities for future work rather than as definitive evidence of systematic bias in PACT performance.

### Limitations and future work

While the results have shown that the PACT can generate reasonable approximations of prosthetist-fabricated sockets, this study also has some limitations.

First, this was a single-site study at a pediatric specialty hospital and included only two prosthetists, so local workflows and case mix may limit generalizability (only three-quarter Northwestern-style sockets were considered). Second, the reference library was limited (n = 19) and under-represents certain morphologies (such as long or bulbous residual limbs), which may bias retrieval and scaling. Third, validation focused on geometric agreement and did not include the fabrication and fitting of PACT-generated sockets or collection of patient-reported outcomes; therefore, clinical performance remains to be established in comparative studies. Fourth, shape capture relied on static scans trimmed at the epicondyles, and does not consider soft-tissue properties, factors that can influence design decisions. Finally, we did not include whole-body anthropometrics (height, weight, BMI) in the analysis or algorithm. The current study focused on limb-level geometry directly used by the PACT; future studies should systematically collect standardized anthropometric data to determine whether incorporating these factors into PACT further improves prediction accuracy.

In light of this, critical future steps include expanding the reference library with data from other hospitals, accounting for differing clinical practices and socket types. In addition, a clinical study that fabricates and fits PACT-predicted sockets and compares them with conventional sockets is needed to confirm patient-reported comfort and function and to support broader implementation in both traditional clinical settings and remote care.

## Conclusion

This paper introduces the PACT, a case-guided retrieve-and-refine pipeline that quickly generates first-draft transradial sockets directly from a single 3D limb scan. By comparing the PACT-predicted and prosthetist-fabricated sockets for 19 participants, we quantified their global and local shape agreement and identified concrete directions for algorithm refinement. The findings indicate that the PACT is able to generate socket geometries sufficiently close to clinical practice for use as a first-draft, which has the potential to eliminate clinician effort during the initial stages of design. The local shape analyses identified where manual expertise and clinician intervention matter the most, particularly around trimlines and anterior–posterior compression, providing clear guidance for rule-based or measurement-informed updates to the PACT.

Future work will focus on expanding the reference library to cover more limb shapes, incorporating soft-tissue and age-related adjustments, and conducting clinical trials to confirm patient-reported comfort and function. In short, the PACT offers a pathway to faster, more consistent transradial socket design while retaining prosthetist expertise for final, patient-specific refinement.
