# Correction: PACT: A practice-driven predictive algorithm for customized transradial prosthetic socket design

**DOI:** 10.1371/journal.pone.0343930

**Published:** 2026-02-27

**Authors:** Vishal Pendse, Calvin C. Ngan, Elaine Ouellette, Neil Ready, Jan Andrysek

The Funding statement for this article is incorrect. The correct Funding statement is as follows: “J.A. received funding from the Natural Sciences and Engineering Research Council of Canada (NSERC) Alliance Grant (No. 571074-21), Holland Bloorview Foundation, and The War Amps. V.P. received funding from the Ontario Graduate Scholarship and Kimel Family Graduate Student Scholarship in Paediatric Disability. The funders had no role in study design, data collection and analysis, decision to publish, or preparation of the manuscript.”
